# On the Dangers Arising from Syphilis in the Practice of Dentistry

**Published:** 1890-10

**Authors:** L. Duncan Bulkley

**Affiliations:** Attending Physician to the New York Skin and Cancer Hospital, etc.


THE
AMERICAN JOURNAL
—OF—
DENTAL SCIENCE.

VOL. XXIV. BALTIMORE, OCTOBER, 1890. No. 6.

ARTICLE I.

### ON THE DANGERS ARISING FROM SYPHILIS IN THE PRACTICE OF DENTISTRY.

BY L. DUNCAN BULKLEY, A.M., M.D.,
Attending Physician to the New York Skin and Cancer Hospital, etc.

Read before the New York Odontological Society, April, 1890.

Happily the recorded instances of the communication of syphilis in connection with the practice of dentistry are relatively few in number when compared with the very numerous cases on record where the disease has been acquired through other innocent channels; for, it must be remarked, the number and variety of modes by which this disease has been spread innocently from one person to another, entirely without sexual transgression, is manifold greater than could be supposed or imagined by one who has not investigated or given some attention to the matter.

The subject of the innocent transmission of syphilis is a very large one, and one to which the public health officials might well direct their attention; but the present time we can consider only a very small and limited subdivision of it,—namely, as the existence of the disease may in any way bring danger through or to any one in the practice of a single branch or specialty in surgery,—that of dentistry.

Although, as before stated, the reported instances where syphilis has been communicated in connection with, or during the operations of dentistry, are relatively few, nevertheless, there are a sufficient number of cases on record not only to show clearly that this unfortunate accident has repeatedly occurred, and may readily happen, but also to direct our attention to the methods or channels through which this may take place, and so to indicate the means by which the danger may be escaped or avoided. To develop these points will be our task this evening.

It would be quite out of place in this assembly to attempt fully any consideration of syphilis as a disease, or even to give a description of its different manifestations and effects, which are more varied and manifold than those of any other known malady. But before entering upon our subject proper, it may be well to briefly recapitulate the points which are well established in regard to the nature and pathology of syphilis, in order that the real dangers arising from the disease may be better understood, with the reasons therefor.

Syphilis is no longer to be looked upon with the utter abhorrence with which it has been regarded in times past, when it was always believed to be the result of sexual transgressions; it is not, indeed, to be considered always as a venereal disease, for advancing knowledge has revealed, and science recorded, thousands of cases where it has been acquired in scores of ways, where the unfortunate victim was as innocent as is one who catches small-pox, scarlatina, or measles; but, of course, the fact still remains that in the enormous majority of instances syphilis is acquired in sexual intercourse, because here is offered the greatest opportunity for abrasions to occur, through which the poison may gain entrance. But, on the other hand, hundreds, or even thousands, of physicians themselves and midwives, have contracted syphilis in the practice of their calling; in numbers of instances it has been conveyed in vaccination, tattooing, cupping, and breast-drawing, and, in fine, there is no end to the curious and previously-unexpected methods by means of which this disease has been innocently communicated from one person to another.

This innocent transmission of syphilis has also occasionally happened in the practice of the various departments of medicine and surgery. Not only have midwives acquired the disease in the practice of their calling, but several small epidemics are on record where a number of women, and from them their children and others, have acquired syphilis from a chancre on the finger of the woman who had delivered them. The disease has also been communicated in various surgical operations, and a striking illustration is found in the history of Eustachian catheterization, where as many as sixty cases were traced to the practice of one person. Instances will be given later where it has occurred in some of the operations of dentistry.

Syphilis is a well-defined disease, depending always upon the entrance of a definite, specific poison, which has never been perfectly isolated, and of whose exact nature we know nothing. It is most probably due to a micro-organism, but although this has been thought to be discovered on several occasions, it has never been satisfactorily demonstrated, nor have inoculations been made with success by any pure cultivation of the same. It suffices for our purpose, however, to recognize that there is a poison, capable of entering the system, and thereby causing syphilis, which can reproduce or multiply itself there, and again, under proper conditions, communicate the disease to all who are properly and sufficiently exposed to its influence.

The poison always enters the system at some definite point, and at this place, generally within from two to four weeks, a local sore, termed a chancre, develops, which is the first external sign of the syphilitic invasion. This sore presents quite different appearances under different circumstances and in various localities, and time fails to attempt to describe these; their appearances may be learned from many recent text-books and journal articles. The only exception to this mode of entrance of the disease is found in the case of hereditary syphilis, where the poison enters with the life, and possibly in some other rare conditions, which need not be entered upon here.

For the entrance of the syphilitic virus a broken epithelial or epidermal surface is necessary, although apparent exceptions to this rule have been occasionally met with. But in some way the poison must come in contact with the absorbing elements of the body,—either blood-vessels or lymphatics,—and by them be taken into the circulation, where it multiplies and produces lesions in various parts of the body, and then probably increases also in the tissues. When once the virus has gained entrance, the individual is syphilitic, and unless the disease is checked by treatment, it will go on to produce, its manifestations, even for many years.

The period during which syphilis is actively inoculable has never been definitely determined, and will probably never be accurately known. It is certainly very contagious, under proper circumstances, during the first year, and also for many months thereafter, and cases are on record where infection has occurred from cases many years advanced in syphilis; indeed, the point or period has never been determined when danger ceases. Although it is questionable if syphilis is often communicated by patients five years after their infection, no prudent man would ever take a shadow of a chance of personal inoculation at this or even at a very much later period. Treatment, of course, greatly modifies the infective power of the disease, and under the fullest possible measure of proper treatment the contagious character of syphilis is greatly lessened, if not entirely destroyed, in some cases, even in the earlier stages of the disease. But all these "ifs" and qualifying statements only show what a dangerous and treacherous affection we have to deal with, and how difficult it is always to be certain of immunity from danger.

The poison of syphilis may be received from four different sources,—1, the initial sore or chancre; 2, from mucous patches; 3, from syphilitic ulcerations; and 4, from the blood. These we will briefly consider in above order.

1. The chancre, or initial sore of syphilis, is occasionally found upon the lips, tongue, and other portions of the buccal cavity, but generally the lesion is so marked and painful that the patient avoids the dentist, and there is relatively little danger of infection from this source. But, on the other hand, this danger sometimes occurs, as in the instance of the case which fell under my own observation, about to be described, where the gentleman, supposing that the chancre was only a local sore, due to sharp and rough teeth, went to his own dentist, and had them filed off, even when the ulcer was very painful and giving off an abundant, virulently contagious secretion; so that his dentist must certainly have been exposed to the same. It is well, therefore, in the case of doubtful sores about the lips, tongue, or mouth, either to be assured of their harmless nature or to exercise such precautions as will insure perfect protection to self and others, which will be considered later.

2. The second source of the contagion of syphilis—namely, mucous patches—is far more fruitful of infection, and that against which special care must be exercised. It is to be remembered that at one time or another these lesions appear in a greater or less amount on the buccal mucous membrane of almost every case of syphilis, so that at some moment or other almost every case is capable of communicating the disease from this source of contagion.

Mucous patches are slightly raw surfaces, of various sizes and shapes, which are at first elevated slightly, and then may become depressed by the loss of the epithelial covering. When newly developed, they are of a redder color than the normal mucous membrane, but later may become of a grayish white. They are always superficial lesions, and often do not cause much annoyance, so that the patient may readily attend to all the duties of life, and may go through considerable dental manipulation while having an abundant crop of mucous patches on the tongue, lips, or buccal cavity. The secretion from them is sticky and intensely contagious. It is from these lesions that fresh chancres are most commonly contracted, and it is this secretion which, adhering to instruments and articles of use or convenience,—such as cups, spoons, pipes, etc.,—commonly gives rise to syphilis in most unexpected manners.

3. Certain deeper ulcerations of syphilis may sometimes give rise to contagion, especially when they occur in the earlier stages of the disease; but practically very few instances of contagion are ever from this source, although this danger should always be guarded against as well.

4. The fourth source of syphilitic infection—namely, the blood—is the least likely to present dangers in connection with dentistry. It is, however, quite possible for blood, which is drawn during an operation or by accident, to communicate syphilis, if it chances to find a proper opportunity to enter another individual. It is just the uncertainty in regard to the possibilities of infection which gives to our subject such great practical interest. In few, if any, of the dozens of methods by which the disease has been innocently transmitted from one person to the other was the possibility of such an accident known, or even suspected, beforehand.

We will now consider some of the observed facts in regard to the communication of syphilis in dentistry, and afterwards examine the modes of transmission and the means of prevention. Our clinical study will naturally divide itself into two lines of thought,—1, in regard to the dangers from syphilis to *patients* undergoing dental operations; and 2, in regard to dangers to the *operator* from the same source.

1. First as to dangers to the *patient* from exposure to the syphilitic poison during dental operations.

Inasmuch as it presents many points of interest, relating both to the patient and operator, I may be allowed first to recite the case alluded to, which came under my own observation and treatment, and which first called my attention particularly to the subject.

Mr. X. W., a gentleman of intelligence and position, aged 60 years, came to me September 11, 1884, on account of a sore on the tongue, which he feared to be a cancer. The history was, that some ten weeks before his first visit, he had first noticed a little point of soreness, which had gradually increased in size, in spite of treatment, until latterly it had come to give him considerable annoyance, so that he was conscious of its presence at all times; the true nature of the sore had evidently not been recognized.

On examination, there was found on the right side of the tongue, about an inch from its tip, a hard, inflamed mass, nearly half an inch in diameter, the centre ulcerating and the edges somewhat everted; it was not painful except when irritating food or drink touched it. The two upper molars were found to have sharp and rough edges, and he had been wearing a red rubber plate until recently. There was a small and painful gland beneath the jaw on that side, slightly enlarged.

Thinking that the ulcer might possibly be due to irritating local causes, he was given a soothing mouth-wash, and an alkali internally. Five days later there was a marked improvement in its condition; the ulcer had a less angry look, but its edge was more clearly defined as the inflammatory element had somewhat subsided. He had been, of his own accord, to his regular dentist, and had had the roughened teeth made smooth, and had left out his set of artificial teeth.

From a careful second study of the case, I then felt convinced that the sore was a chancre, a primary lesion of syphilis, and he was immediately put on antisyphilitic treatment; the general eruption and other symptoms which followed a few weeks later rendered the diagnosis certain, together with the remarkable manner in which the sore healed and symptoms vanished under the proper treatment for syphilis.

In searching for the mode by which the syphilitic poison had gained entrance, it was learned that, during the month or so previous to the appearance of the sore upon the tongue, he had, through the persuasion of a friend, been under the care of a dentist of the cheaper, advertising order, who, he had noticed, was not at all cleanly either in his person or with his instruments. He could not locate the exact date of the injury of the tongue by the dental instruments, but work had been done in that locality, and he remembered the instrument occasionally slipping, as will often happen, inflicting injury to the soft parts. He was a married man with a family, and was very desirous of learning how he had become infected; he had certainly not been exposed in sexual intercourse, nor in any other manner which we could discover.

The interesting points in the case are: First, that while the proof is not absolute that he was infected in the dentist's chair, still the circumstantial evidence is so strong that little if any doubt can be entertained that the poison came through this channel. The habits and ways of the particular dentist were such that poisonous material from the mouth of a previous syphilitic patient could readily have been transferred on instruments or otherwise to the wound made in the tongue, either by the sharp teeth or by a slip of an instrument. The second interesting point is that this patient, before the true nature of the disease was ascertained, had been to his own regular dentist for smoothing the teeth, and so had certainly exposed him, and others through him, to the poison, which was secreted freely from the raw surface of the chancre.

The earliest recorded cases of the transmission of syphilis in dental operations are in connection with the transplantation of teeth, during the last quarter of the eighteenth century.

Sir William Watson * published a case of this de scription, and John Hunter † relates two similar cases about which there can be no doubt. J. C. Lettsom ‡ also recorded three cases: of these one was personal, one seen by a Dr. Hamilton, and the third occurred in America, having been observed by Kuhn in Philadelphia; these gentlemen furnished notes of the cases to Dr. Lettsom. This mode of transmission does not occur again in Literature, to the knowledge of the writer, although Gibier § says that "Cases have been recently related." In view, however, of a recent revival of the operation of tooth transplantation, or implantation, it is quite possible that the future may furnish fresh instances of this mode of the innocent acquiring of syphilis.

From this period no other causes of the transmission of syphilis through dental procedures are found recorded for nearly a century; indeed, not until the advent of modern operative dentistry and active medical observation.

The first case met with is one reported by Dr. C. W. Dulles, * of Philadelphia, and which was also seen by the late Dr. Maury. The patient, a female domestic, of excellent character, developed a chancre of the lip two weeks

* Watson, "Transactions of College Surgeons," 1785, iii. p. 328.

† Hunter, "Treatise on the Venereal Disease," 1st Engl. ed., 1786; 1st Amer. ed., Phila., 1818, p. 362.

‡ Lettsom, *Transactions Lon. Med. Soc.*, vol. i., 1787, p. 137.

§ Gibier, "Ann. de Dermat. et de Syph.," 1882, p. 129.

* Dulles, *Phila Med. and Surg. Reporter*, Jan. 1878.

after a visit to a dentist; on that occasion he extracted a tooth, and later did some cleansing of the teeth. Although no confirmation was obtained, it seemed reasonable to suppose that the operation of extraction was in some way responsible for the inoculation.

Dr. F. N. Otis † also mentions a chancre of the lip which occurred in a gentleman "about three weeks after a morning spent in a dentist's chair."

Lancereaux ‡ relates a similar case of chancre of the lower lip in a woman, after extraction of a tooth and other dental work, and Giovannini, § of Bologna, has reported a chancre of the lip apparently from a dentist's instrument.

Leloir * mentions having seen a man with chancre of the gum, in whom the infection seemed to have taken place in consequence of cleaning and filling a cavity in a tooth with soiled instruments. Lydston † has likewise reported the case of a woman with syphilis, in whom the chancre on the gum, below the lower middle incisors appeared to be the result of some cleaning and repairing of the teeth done three weeks previously; the glands beneath the jaw were enlarged, beginning a week or more after the appearance of the sore on the gum.—*International Dental Journal.*

---

ARTICLE II.

### THE HYPNOTISM CRAZE AND ITS DANGERS.

Hypnotism, which is now at the height of fashion, is nothing more than a new edition of the threadbare practice of mesmerism somewhat revised, enlarged and greatly im-

† Otis, "Lectures on Syphilis," New York, 1881, p. 102.
‡ Lancereaux, "Proc. Acad. de Med. de Paris," *Union Med.*, 1889, xlviii. p. 655.
§ Giovannini, "Le Sperimentale," 1889, p. 262.
* Leloir, "Lecon sur la Syphilis," 1886, p. 62.
† Lydston, *Journ. Amer. Med. Assoc.*, 1886, vi. p. 654.

